# Photocrosslinkable liver extracellular matrix hydrogels for the generation of 3D liver microenvironment models

**DOI:** 10.1038/s41598-021-94990-z

**Published:** 2021-07-30

**Authors:** Akhilandeshwari Ravichandran, Berline Murekatete, Denise Moedder, Christoph Meinert, Laura J. Bray

**Affiliations:** 1 Institute of Health and Biomedical Innovation, Queensland University of Technology (QUT), Kelvin Grove, Australia; 2 ARC Training Centre for Cell and Tissue Engineering Technologies, Queensland University of Technology (QUT), Kelvin Grove, Australia; 3 Science and Engineering Faculty, School of Mechanical, Medical and Process Engineering, Queensland University of Technology (QUT), Brisbane, Australia; 4 Herston Biofabrication Institute, Metro North Hospital and Health Service, Herston, Australia

**Keywords:** Biomedical engineering, Biomaterials

## Abstract

Liver extracellular matrix (ECM)-based hydrogels have gained considerable interest as biomimetic 3D cell culture environments to investigate the mechanisms of liver pathology, metabolism, and toxicity. The preparation of current liver ECM hydrogels, however, is based on time-consuming thermal gelation and limits the control of mechanical properties. In this study, we used detergent-based protocols to produce decellularized porcine liver ECM, which in turn were solubilized and functionalized with methacrylic anhydride to generate photocrosslinkable methacrylated liver ECM (LivMA) hydrogels. Firstly, we explored the efficacy of two protocols to decellularize porcine liver tissue using varying combinations of commonly used chemical agents such as Triton X-100, Sodium Dodecyl Sulphate (SDS) and Ammonium hydroxide. Then, we demonstrated successful formation of stable, reproducible LivMA hydrogels from both the protocols by photocrosslinking. The LivMA hydrogels obtained from the two decellularization protocols showed distinct mechanical properties. The compressive modulus of the hydrogels was directly dependent on the hydrogel concentration, thereby demonstrating the tuneability of mechanical properties of these hydrogels. Immortalized Human Hepatocytes cells were encapsulated in the LivMA hydrogels and cytocompatibility of the hydrogels was demonstrated after one week of culture. In summary, the LivMA hydrogel system provides a simple, photocrosslinkable platform, which can potentially be used to simulate healthy versus damaged liver for liver disease research, drug studies and cancer metastasis modelling.

## Introduction

The liver plays a crucial role in many biological functions such as metabolism, homeostasis, detoxification, bile production, electrolyte regulation and immunity. Despite its self-regenerative capacity, liver-related diseases such as drug-induced liver injury, fatty liver disease, hepatitis, fibrosis, cirrhosis and hepatocellular carcinoma, affect a major population across the world and result in significant healthcare burden^1^. Often, these pathological states are accompanied by elevated liver tissue stiffness^2^ which is a key physical property which governs hepatocyte proliferation, phenotype^3,4^ and migration^5^. Conventional laboratory models used to study these pathologies are based on hepatocyte cultures propagated in monolayer or whole liver explants. However, these approaches lack physiological relevance or pose significant problems of batch-to-batch variability, thereby severely limiting their translational value. This has prompted the development of more controlled three-dimensional (3D) in vitro liver models that closely mimic the liver microenvironment and improve the understanding of liver pathologies and develop targeted therapies^6–8^.

To recapitulate the native cellular microenvironment, tissue engineering strategies have successfully utilized decellularized extracellular matrix (ECM) for in vitro tissue generation^9,10^. With a distinct 3D structure, organization and function, tissue-specific ECM plays a crucial role in governing cellular dynamics, communication, tissue integrity and function^11^. Incorporation of these tissue-specific ECM components in 3D models systems can therefore aid in achieving tissue generation with enhanced physiological relevance^12^. One of the most commonly used strategies for obtaining decellularized ECM involves chemical agents such as ionic and non-ionic detergents, acids/bases, alcohols, and chelators^13^. While all the above agents essentially aid in removing cellular content from the cells, their efficiency varies with tissue density and they can alter the properties of the ECM in different ways. While non-ionic detergents like Triton X-100 are gentle and largely preserve the native ECM protein structure, ionic detergents such as SDS are stronger and more effective, but may denature proteins^13^. Therefore, a single detergent may not be sufficient in all circumstances. Specifically for liver tissues, a wide variety of decellularization techniques have been used to produce ECM scaffolds^14–17^ or to prepare solubilised ECM which is used to coat tissue culture plastic^18–22^, as media additives^23,24^, in sandwich cultures^25,26^, and as ECM hydrogels^21,22,27–33^ to improve long-term maintenance of hepatic functionality. Predominantly, studies have used solubilized liver ECM to create collagenous hydrogels fabricated by thermal gelation, resulting in a biomimetic microenvironment for the cells to attach and grow^21,22,27–33^. Yet, despite several related studies published in the last decade^22,27,31–35^, there is little consensus on the most effective decellularization method to generate liver ECM hydrogels with relevant biochemical and biomechanical properties.

Further, the application of commonly used self-assembled ECM hydrogels is limited by their slow and largely uncontrolled gelation, as well as non-physiological mechanical properties^36^. Current ECM hydrogel-based liver tissue models offer little control of their physical properties^2,37,38^ and have not sufficiently considered the stiffness of hydrogels and its correlation with liver physiology and pathology^39^. Functionalization of natural polymers using photocurable components has been employed as a technique for rapid manufacture of hydrogels with controlled gelation^40–47^. By amalgamating the advantages of the natural polymers, like biocompatibility and degradability, along with reproducible physicochemical properties imparted by chemical functionalization, these resulting hydrogels can generate controllable, reproducible, and relevant 3D tissue models^40–46^. Yet very few studies have attempted to modify the liver ECM hydrogels to improve their physicochemical properties^25,48–50^.

In this study, we report the efficiencies of two decellularization protocols for porcine liver developed to study the combinatorial and temporal effects of commonly used chemical agents such as SDS, Triton X-100 and ammonium hydroxide^15,31^. The decellularized liver tissues obtained were solubilized and methacrylated to produce LivMA (methacrylated, solubilized liver ECM), which formed stable hydrogels upon photocrosslinking^51–53^. Functionalization allowed the manufacture of LivMA hydrogels with reproducible and improved physical properties^54,55^. The LivMA hydrogels were characterized for their degree of functionalization, mechanical properties and cytocompatibility.

## Results

### Optimization of decellularization of porcine liver tissues

**Figure 1:**
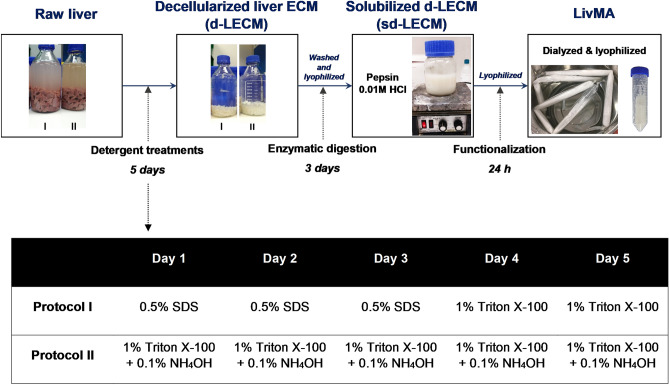
Experimental workflow for obtaining LivMA (Liver ECM methacryloyl): Raw liver tissues were minced finely, rinsed in ultrapure water followed by a 5-day detergent treatment to obtain decellularized liver ECM (d-LECM) using decellularization protocols I and II. The d-LECM samples were enzymatically digested for 3 days using pepsin followed by methacrylation of the solubilized d-LECM, dialysis and lyophilization to obtain LivMA.

With an aim to develop photocrosslinkable, tuneable and reproducible liver ECM hydrogels from porcine liver tissues, we adapted methods from protocols previously developed in the group^53^ and followed the steps outlined in Fig. 1 including porcine liver decellularization (detergent-based), enzymatic solubilization (pepsin-based), functionalization (methacrylation), as well as dialysis and lyophilization to obtain LivMA (methacrylated solubilized liver ECM) (Fig. 1). Firstly, we assessed the effects of two detergent protocols, I and II, that involved commonly used decellularization agents including Triton X-100, sodium dodecyl sulfate (SDS) and ammonium hydroxide (NH_4_OH) over a period of 5 days (Fig. 1).

**Figure 2:**
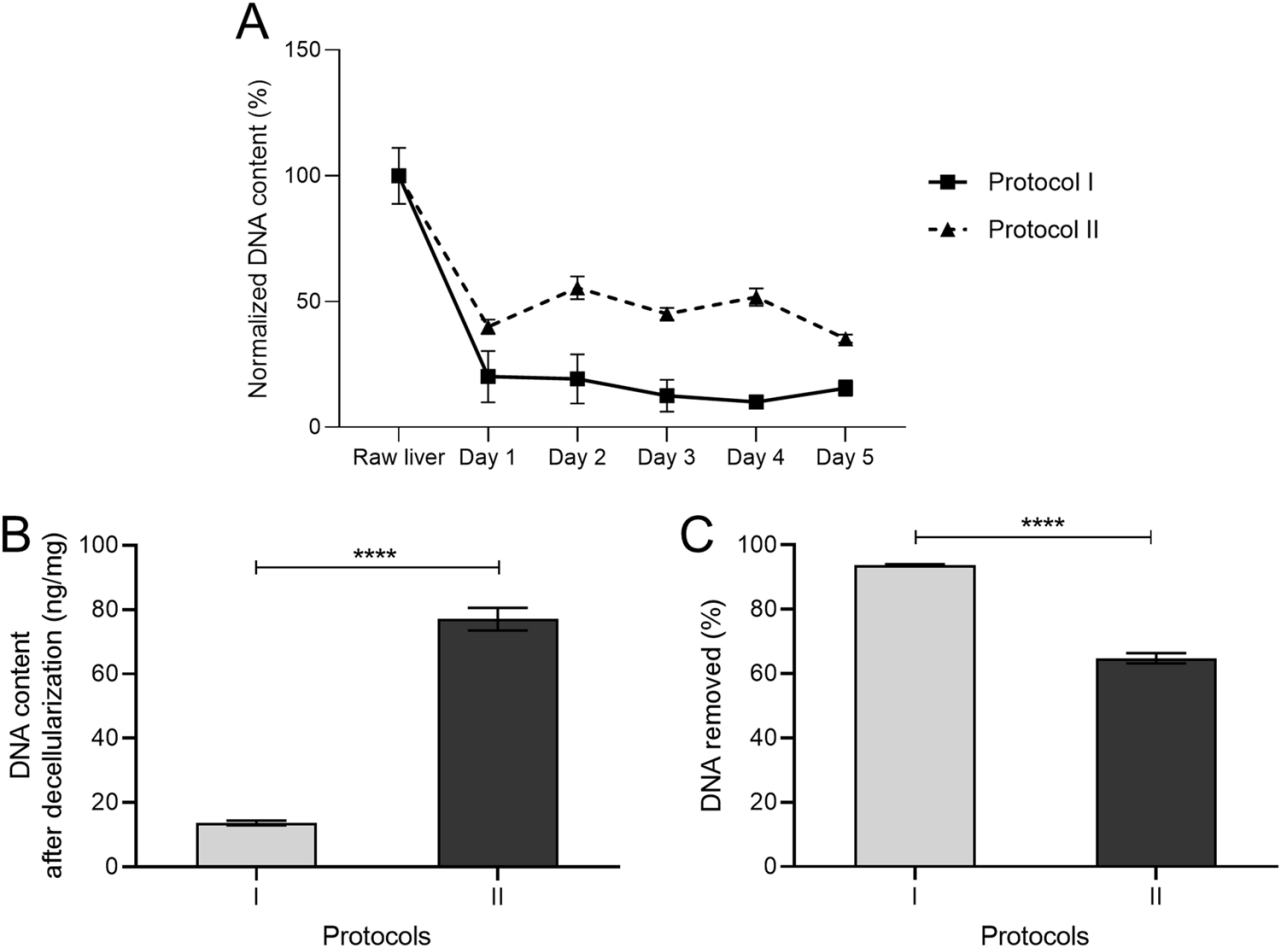
Decellularization of porcine liver tissues using detergent treatment protocols I and II. (**A**) Percentage of DNA content (normalized to raw liver tissues) obtained from tissues treated by both the protocols on each of the 5 days (n = 3, mean ± SD). (**B**) DNA content (n = 3, ng/mg of liver tissue) present in the tissue after the detergent treatments (n = 3, mean ± SD). (**C**) Percentage of DNA removed in each of the protocols (n = 3, mean ± SD).

To determine the decellularization efficacy of the protocols throughout the duration of detergent treatment, we analyzed the DNA content of the tissues each day of the protocol. After the first day of treatment, we observed a ~ fivefold drop in DNA content (normalized to raw liver DNA content) in Protocol I and ~ 2.5-fold drop in Protocol II (Fig. 2A). DNA content sustained at these levels until the end of the 5 days of treatments (Fig. 2A). The DNA content (ng/mg of wet weight of the tissue) after decellularization was 13.7 ± 0.7 ng/mg from protocol I and 77 ± 3.5 ng/mg from protocol II by the end of the treatments (Fig. 2B). This corresponded to a removal of 93.7 ± 0.3% of DNA using protocol I and 64.8 ± 1.6% from protocol II (Fig. 2C). Interestingly, we observed an increase in DNA content on some of the days in protocol II when compared to the previous time point likely due to the sensitivity of the detection method to the detergents in these protocols^56^.

**Figure 3:**
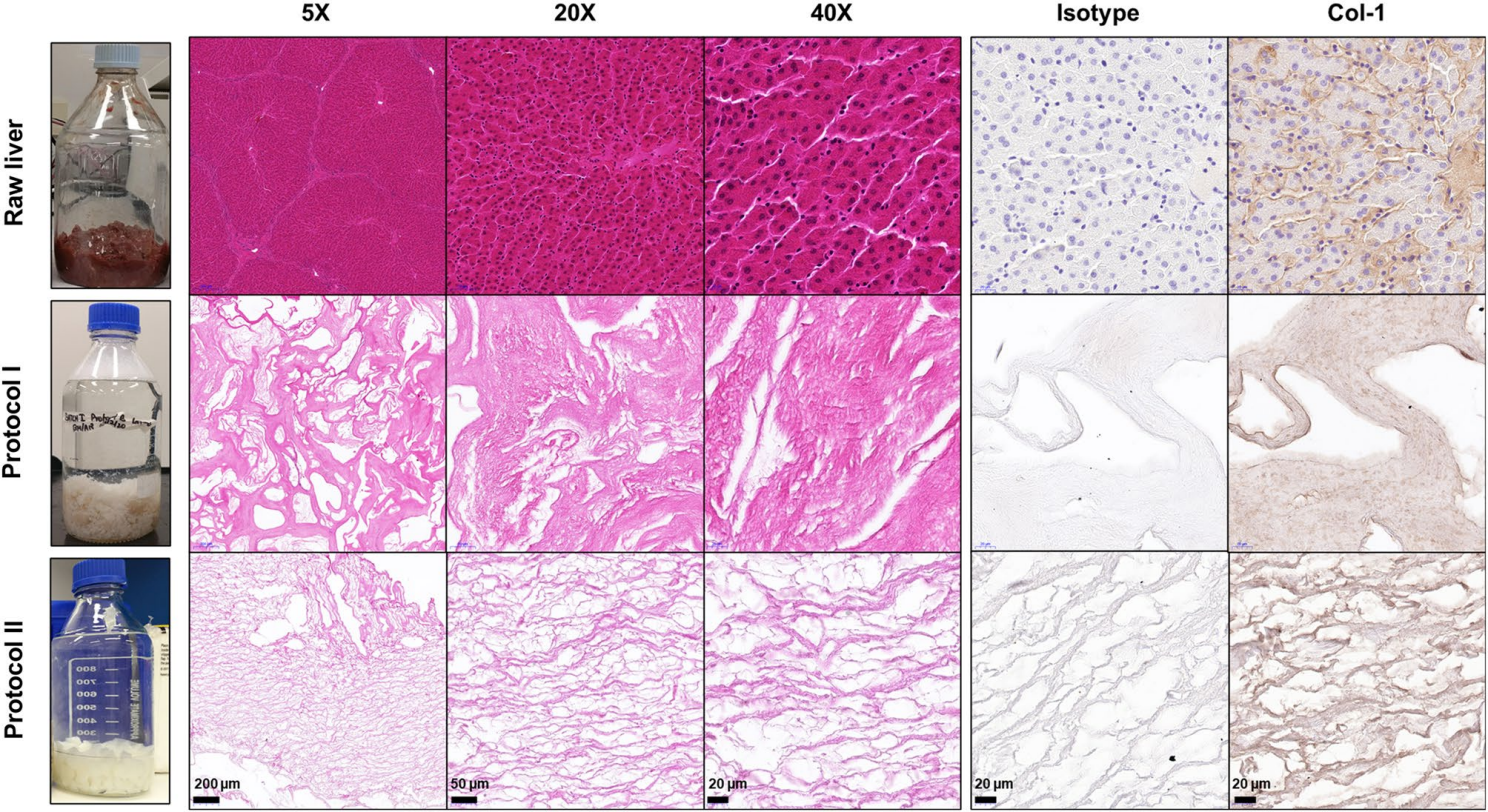
Representative images from H&E staining (5 ×, 20 ×, 40 ×) and immunohistochemical staining for Isotype control and Col-1 (40 ×) of 5 μm thick tissue sections from raw liver tissue and decellularized liver tissue from Protocol I and Protocol II.

To qualitatively visualize the structure and composition of the decellularized tissues from protocols I and II, we used histochemical staining to detect cellular nuclear material and collagen content. Raw liver tissue H&E images revealed intact liver tissue morphology with cells organized in hepatic lobules (Fig. 3). The representative H&E images of the decellularized tissues from protocol I and protocol II showed the loss of native lobular morphology in the decellularized liver tissues with no visible cell nuclei (stained purple), thereby confirming the efficacy of decellularization in these groups (Fig. 3). Remnant nuclear content was sparsely distributed across the sections in the decellularized tissues from both the protocols (Supp Fig. 1). Stronger eosinophilic staining was observed in decellularized tissues from protocol I when compared to protocol II. Immunohistochemical staining was performed to determine the presence of collagen type I in the decellularized liver tissues. Results revealed that the ECM collagen was preserved post-decellularization in both protocols I and II. The decellularized tissues from protocol II presented gaps between the thick fibrillar networks when compared to the more continuous structure observed in protocol I.

### Solubilization and methacrylation of decellularized liver ECM

**Figure 4:**
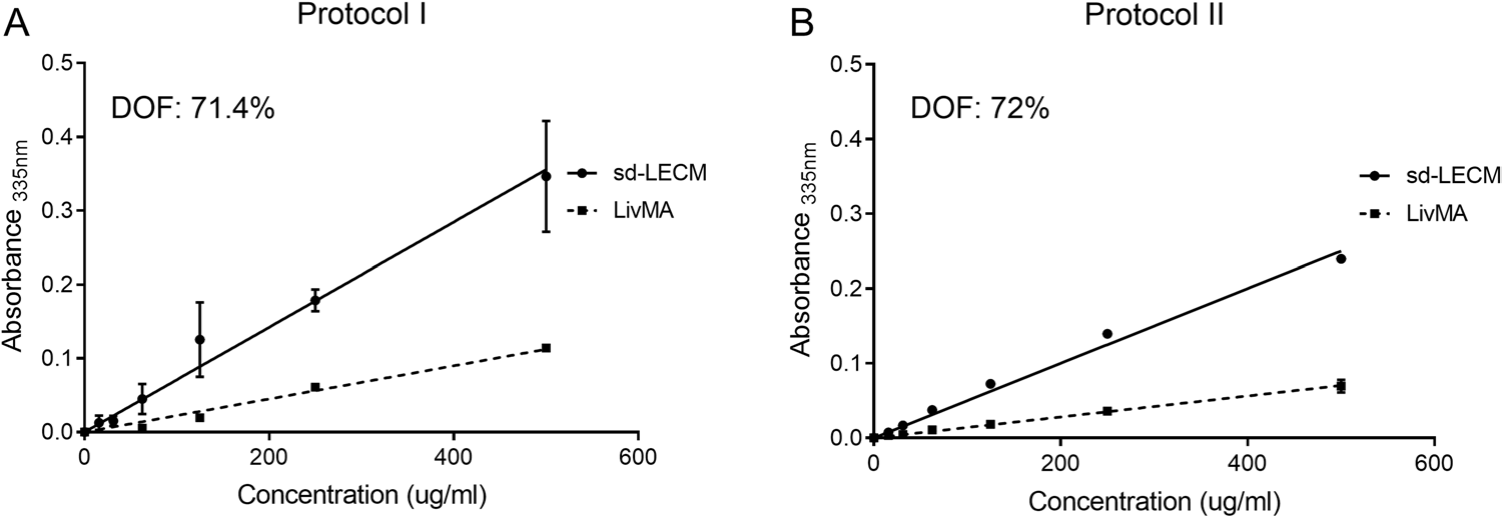
Degree of amine functionalization (%) assessed using a TNBS assay. (**A**, **B**) Slope obtained from the range of concentrations versus absorbance from functionalized samples (LivMA) compared to that from the non-functionalized solubilized, decellularized liver ECM (sd-LECM) samples for protocol I and protocol II (n = 3–6, mean ± SD).

The decellularized tissues were lyophilized, solubilized using pepsin and 0.1 M HCl to produce solubilized d-ECM (sd-LECM) from protocols I and protocol II. This was followed by functionalization, dialysis, and lyophilization to produce LivMA. To determine the efficiency of methacrylation, the degree of functionalization (DOF) was calculated from the slope of the linear regression lines obtained from unmethacrylated samples (sd-LECM) and methacrylated samples (LivMA) using a TNBS assay^57^. Protocol I produced LivMA gels with a DOF of 71.4% (n = 3) (Fig. 4A) and protocol II LivMA gels had a DOF of 72% (n = 6) (Fig. 4B).

### Photocrosslinking of LivMA hydrogels

Prior to photocrosslinking of LivMA, we attempted to make hydrogels by spontaneous thermal gelation at 37 °C in the absence of photocrosslinker from sd-LECM and LivMA. The sd-LECM derived from protocols I and II did not gel via thermal gelation at 37 °C. At the concentrations we had used (0.5–1.75%), the LivMA from protocol I did not show any thermal gelation at 37 °C even after 6 h of incubation. LivMA from Protocol II did not show thermal gelation at lower concentrations and formed soft hydrogels at concentrations > 1% (1.5%: 1.4 ± 0.3 kPa, 1.75%: 2.4 ± 0.25 kPa) (Supp Fig. 2) after 30 min of incubation at 37 °C. Beyond these concentrations, uniform dissolution of the stock was found to be difficult and hence we retained our stock concentrations for LivMA at 1.75%.

**Figure 5:**
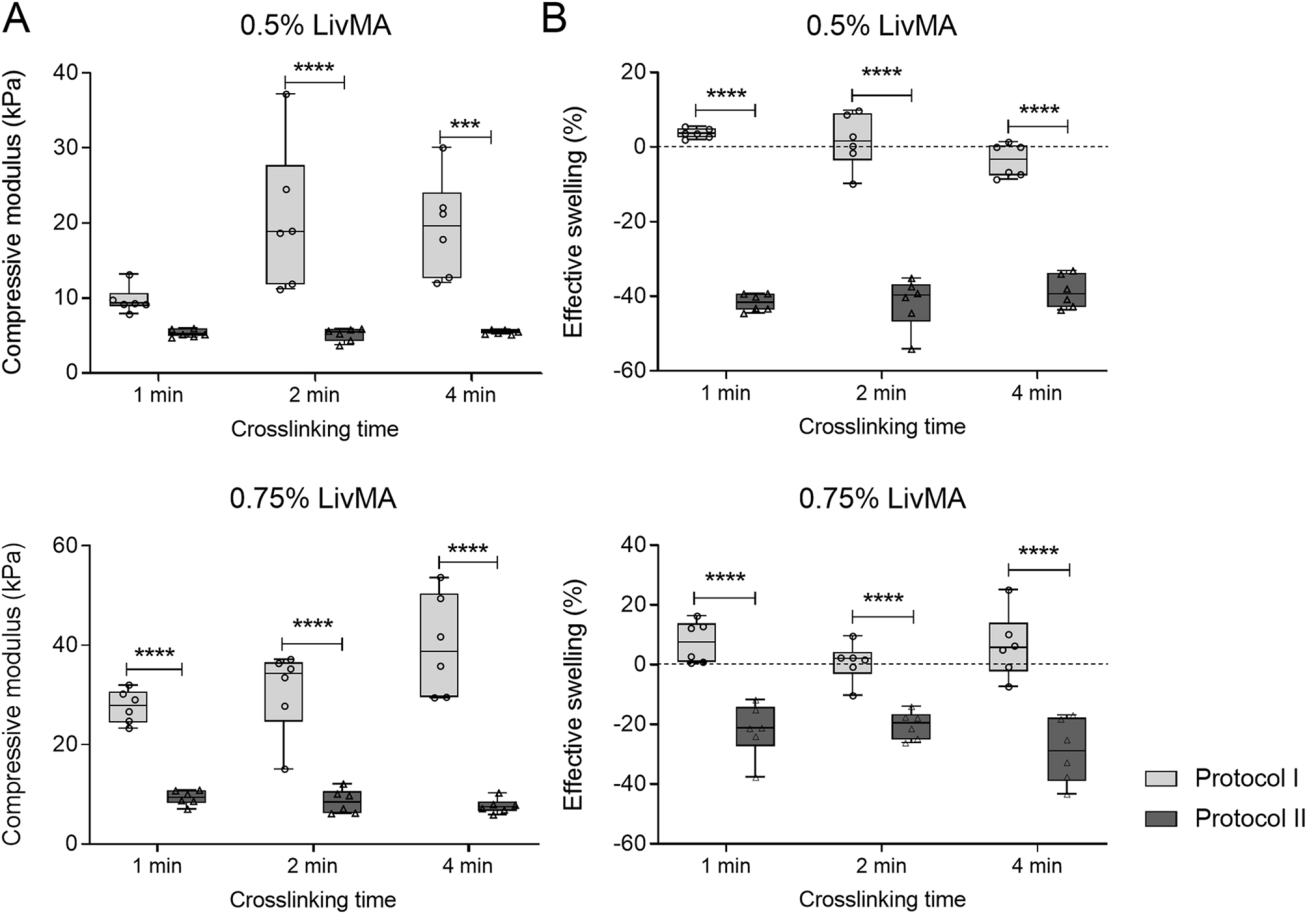
Mechanical characterization of LivMA hydrogels derived from protocol I and protocol II. (**A**) Box plot (10th–90th percentile) showing the compressive moduli of hydrogels from protocol I and II at 0.5% (w/v) LivMA and 0.75% (w/v) LivMA with different crosslinking times—1 min, 2 min, 4 min (n = 6, line at median). (**B**) Box plot (10th–90th percentile) of the effective swelling (%) of hydrogels calculated by weighing the hydrogels before and after swelling in PBS overnight at 37 °C (n = 6, line at median).

Preliminary studies for photocrosslinked LivMA gelation from both protocol I and protocol II showed successful formation of stable, optically clear, and easy-to-handle LivMA gels at concentrations of ≥ 0.5% (w/v) and crosslinking times of ≥ 1 min (data not shown). We investigated the mechanical properties of these hydrogels using compression testing and effective swelling. The compressive moduli for hydrogels obtained from protocol I revealed a wide range from 9.8 ± 1.8 kPa to 39.9 ± 10.1 kPa (Fig. 5A). In comparison, the hydrogels obtained from protocol II had lower stiffnesses in all the crosslinking times and concentrations. The moduli for protocol II hydrogels ranged between 5.1 ± 0.9 kPa and 9.3 ± 1.5 kPa (Fig. 5A). From both the protocols, we observed a significant dependence of the compressive modulus of the LivMA hydrogels to the concentration with a ~ 1.4 to ~ 2.8-fold increase in the modulus with an increase in LivMA concentration from 0.5% (w/v) to 0.75% (w/v) (n = 6, *p* < 0.0001). This demonstrated the control of hydrogel stiffness by modifying the concentration of the LivMA hydrogels. Between the protocols, increasing crosslinking times from 1 to 4 min resulted in an increase in the compressive modulus for gels from protocol I. No such effects were observed for the gels from protocol II with crosslinking times.

With respect to the swelling kinetics, we did not observe a defined trend with the crosslinking times for both 0.5% (w/v) LivMA and 0.75% (w/v) LivMA obtained from both the protocols (n = 6) (Fig. 5B). Interestingly, hydrogels derived from protocol II exhibited a strong negative swelling behavior, while hydrogels from protocol I did not. This shrinkage effect was higher (twofold) in the 0.5% (w/v) LivMA hydrogels (protocol II) compared to the 0.75% LivMA hydrogels (protocol II) (n = 6, *p* < 0.0001).

### Cell-encapsulation in LivMA hydrogels

**Figure 6:**
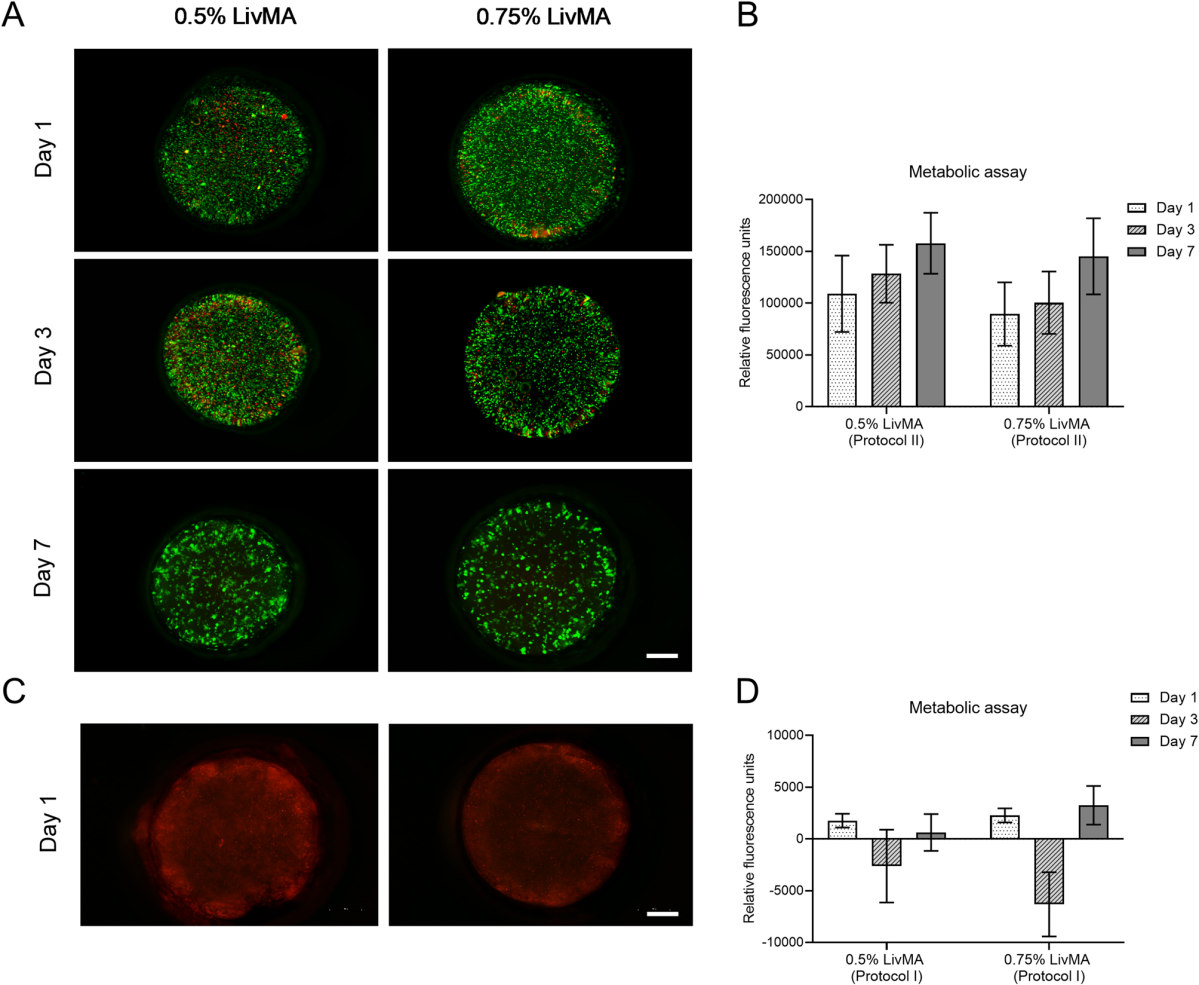
Cytocompatibility of 0.5% (w/v) LivMA and 0.75% (w/v) LivMA hydrogels. (A) Live/Dead imaging of cell-laden LivMA hydrogels derived from protocol II on days 1, 3 and 7 (green cells: live, red cells: dead) (scale bar: 1 mm). (B) Metabolic assay of cell-laden LivMA hydrogels (protocol II) on day 1, day 3 and day 7 (mean ± SD, n = 3). (**C**) Live/Dead imaging of cell-laden LivMA hydrogels derived from protocol I on day 1 (green cells: live, red cells: dead) (scale bar: 1 mm). (**D**) Metabolic assay of cell-laden LivMA hydrogels (protocol I) on day 1, day 3 and day 7 (mean ± SD, n = 3).

Cytocompatibility of the hydrogels from protocol I and protocol II was assessed using a live/dead assay and a metabolic assay. For the hydrogels from Protocol II, we observed viable cells over the duration of one week and the cells aggregated to form spheroidal structures by day 7 in 0.5% (w/v) LivMA and 0.75% (w/v) LivMA that were photocrosslinked for 1 min (Fig. 6A). Quantitative results from the metabolic assay confirmed cell viability and growth in the 7 days of culture (Fig. 6B). Hydrogels derived from protocol I on the other hand were cytotoxic and resulted in cellular death within one day of encapsulation. This was confirmed by both the live/dead assay which showed no viable cells (green) (Fig. 6C) in the hydrogels and the metabolic assay (Fig. 6D). To confirm if the cytotoxic effects of hydrogels derived from Protocol I were a result of the presence of unmethacrylated products, we added sd-LECM (1%) solution to IHH cells propagated in monolayer on tissue-culture plastic. We observed cellular death in the cultures within one day of exposure to sd-LECM from protocol I likely due to residual SDS from the detergent treatments^58,59^ (Supp Fig. 3).

## Discussion

Liver ECM hydrogels obtained using various decellularization methods have been extensively studied for generating engineered liver tissues and in vitro models^21,22,27–33^. However, the gelation kinetics is generally temperature-dependent with little control over the gelation time and mechanical properties of the hydrogels. The present study analyzed the efficiency of two detergent treatment protocols to produce decellularized porcine liver tissues, which were solubilized and functionalized using methacryloyl groups to generate photocrosslinkable LivMA hydrogels. The LivMA hydrogels were assessed for their degree of functionalization, mechanical stiffness and cytocompatibility.

In the last decade, there has been significant interest in the development of ECM-derived materials for engineering in vitro models that can provide better understanding of tissue physiology, pathology and function^60,61^. ECM has been predominantly obtained through animal organ and tissue decellularization using different chemical detergents^62^. This is a critical step because the choice of detergent treatment has been shown to affect gelation kinetics, mechanical properties and biological activity of the resulting ECM hydrogels^35,58,63^. While some studies have reported the use of a single detergent treatment for successful decellularization of liver tissues^27,29,34^, other studies have shown improved efficiency of decellularization by combinational use of detergents^20,22,31^. Many protocols can be potentially developed and tested from the varying concentrations, processing times and combinations of commonly used detergents such as Triton X-100, sodium dodecyl sulfate (SDS) and ammonium hydroxide (NH_4_OH). To determine the ideal decellularization method for our study, we adapted protocols based on previous liver decellularization studies^15,31^. Focusing on parameters that have already been independently assessed for the decellularization of liver tissue enabled us to narrow down the wide range of parameters, compare their decellularization efficiencies and capacities to generate liver-ECM based hydrogels. By exploring the synergistic effects of different combinations of commonly used detergents^15,31^, we successfully reported the decellularization efficiency of porcine livers and used these protocols for further processing into LivMA hydrogels. Our results also showed that the DNA was removed to its maximum extent in the first 24 h of the treatment itself and hence future studies may aim to reduce the duration of the protocols to reduce exposure time of the liver tissues to chemical detergents.

Our next step was to solubilize these dLECM using enzymatic digestion. In our preliminary work, we observed poor enzymatic digestion of the decellularized tissues at 37 °C (data not shown). In accordance with previous studies, however, we achieved complete solubilization at room temperature^30,64^. The majority of previous studies have used unfunctionalized, solubilized liver ECM to encapsulate liver cells by spontaneous gelation at 37°C^22,26,27,31,32,34,35^. While these studies have collectively demonstrated maintenance of liver-specific functions, thermal self-assembly of ECM hydrogels is a largely uncontrolled, time-consuming process which leaves little room to tune key physicochemical matrix parameters such as stiffness and porosity. It has been shown to take between 30 min and 6 h to produce stable hydrogels^22,27,32,34,35^. Further, pure ECM-based hydrogels have been shown to undergo rapid degradation owing to poor mechanical properties^65^. By methacrylation of the solubilized liver ECM in our study, we demonstrate the generation of stable LivMA hydrogels by rapid photocrosslinking (1 min). The photocrosslinked LivMA hydrogels from both the protocols were stiffer than the LivMA hydrogels that were fabricated by thermal gelation. Previous studies have shown that ECM-derived hydrogels could not be formed via spontaneous thermal gelation from decellularization methods that used SDS at certain concentrations^58,63^. Similar to previous studies, the Protocol I LivMA hydrogels did not gel spontaneously at 37 °C. Further, the slow gelation times of pure ECM-hydrogels preclude the manufacture of 3D tissues with distinct and complex geometries to represent the native tissue structure.

Here, the functionalization enables us to generate liver ECM tissues with higher mechanical stiffnesses. This is vital because liver stiffness has been demonstrated to be an effective marker of liver diseases, including liver fibrosis, cirrhosis, fatty liver disease, hepatocellular carcinoma and liver metastasis^2,39,66^. There have been several reports on liver elasticity measurements and the reported stiffnesses have differed depending on the type of technique used^2,66^. Overall, there is a consensus on the normal liver stiffness values to be below 6 kPa and pathological liver tissues including fibrotic, cirrhotic, and cancerous liver tissues to have stiffnesses higher than 8 kPa^2^. Yeh et al. had reported a 2–tenfold increase in liver stiffnesses in fibrotic and cirrhotic liver tissues (~ 20 kPa) when compared to healthy liver tissues^39^. Additionally, tissue stiffness has been shown to have a direct correlation with cancer progression and therapy resistance in solid tumors^67,68^. In a study that characterized the elasticity values of liver tumors, the results reported a ~ 7.5 fold increase in liver stiffnesses (~ 50 kPa) of hepatocellular carcinomas and liver metastases when compared to normal, healthy liver tissues^66^. Hence, it becomes essential to develop robust in vitro models that allow manipulation of tissue stiffness. Few studies have characterized mechanical properties of liver ECM-based hydrogels and the reported stiffnesses are not high enough to represent the pathological liver tissue conditions^32,50^. Lee et al. demonstrated enhanced modulus of rat liver ECM hydrogels (0.27 kPa to 0.4 kPa) by modifying the hydrogel concentrations (10 mg/mL to 20 mg/mL)^32^. Other studies have used crosslinkers like riboflavin^49,69^, N-(3-Dimethylaminopropyl)-N′-ethylcarbodiimide hydrochloride (EDC) and genipin^65^ to inherently crosslink the decellularized ECM and tailor mechanical properties. In our study, methacrylation enabled easy manipulation of the modulus, and generated liver constructs with higher stiffnesses that can be used to mimic both normal liver tissues and pathological liver tissues. Here, we have demonstrated that by increasing the concentration of LivMA, a stiffness as high as 162 kPa (see Supp Fig. 2) could be achieved. By optimizing photocrosslinking parameters, we were able to create hydrogels with a range of stiffnesses to represent the native livers, normal as well as pathological. In addition to hydrogel concentration and crosslinking times, future studies can also modify crosslinker concentrations to achieve hydrogels with varying mechanical properties. Rapid crosslinking and improved mechanical properties can aid in generating desired architectures with the LivMA hydrogels using customized PTFE molds.

The LivMA hydrogels prepared from protocol II supported cellular growth over a period of 7 days. However, this was not the case with protocol I, where we found that the LivMA hydrogels had poor cell viability after 24 h of encapsulation (Fig. 6C). It is unlikely that these cytotoxic effects were related to byproducts of the LivMA synthesis as high levels of cell death were also observed with the unfunctionalized sd-LECM solutions from protocol I. The cytotoxicity was potentially a result of the remnant SDS in the LivMA hydrogels from the Protocol I decellularization. Similar to our study, White et al. found SDS (1%) to be most effective detergent for tissue decellularization and yet resulted in reduced cell numbers with poor morphology as a result of residual SDS on the decellularized tissues^59^. Another study also reported cytotoxicity of SDS-decellularized ECM hydrogels derived from porcine corneas which was contributed to remnant SDS in decellularized tissues^58^. These results along with results from the DNA content analyses indicate that residual detergent may be present in both the decellularized tissues and the ECM hydrogels which can interfere with their characterization and function. Residual detergent present in tissues post decellularization is a commonly reported, yet less frequently addressed problem in tissue decellularization studies. Quality control methods to evaluate the removal of detergent should be incorporated at every step starting right from decellularization to avoid potential cytotoxicity effects observed during the fabrication of hydrogels. Future studies will introduce repeated washing steps followed by relevant physicochemical characterization of the decellularized tissues and the ECM hydrogels^59^, to ensure complete removal of detergents and provide more accurate understanding of the effects of decellularization methods. While we did not observe significant differences in the cellular viability and metabolism between the 0.5% and 0.75% LivMA obtained from Protocol II in the first week of culture, the platform provides scope to analyse the effects of varying stiffnesses in liver disease models. Previous hydrogel studies, although not liver ECM-based, have demonstrated that mechanical stiffnesses higher than normal liver tissue can affect the proliferation, gene responses^70^ and phenotype^71^ of liver cells. Future studies will aim to specifically assess the expression of markers of fibrosis such as procollagen I, collagen III, connective tissue growth factor, TGFβ1 and α-SMA in response to increased stiffnesses of the LivMA hydrogels. The effects of tissue source and donor variability on tissue decellularization and hydrogel properties have not been explored in our study and will need to be investigated in order to standardize the fabrication process^72^. Long-term culture of the cell-laden LivMA hydrogels and its effects on specific hepatogenic properties such as urea and albumin synthesis will also be examined in future studies.

In conclusion, we have reported the efficiencies of two different detergent protocols to form decellularized liver ECM tissues. We have developed a novel, photocrosslinkable hydrogel system that is composed of decellularized, solubilized and functionalized porcine liver ECM. Mechanical tuneability and cytocompatibilty of these hydrogels have been demonstrated. This hydrogel system has the potential for recapitulating healthy and pathological states of liver tissues for future use in drug response studies.

## Methods

### Procurement of porcine liver tissue

The porcine liver tissue was obtained from a local butcher. Fresh tissue was immediately chopped into 1 cm^3^ pieces and washed in distilled water. The liver pieces were washed in distilled water for one week at 4 °C with constant stirring. Samples from fresh liver tissue (< 50 mg wet weight, n = 6) were collected and stored at -80 °C for analysis.

### Generation of LivMA material

#### Decellularization process

The washed liver tissues were decellularized using four different detergent treatments named as protocols I and II (Fig. 1), which included the following chemical solutions at different concentrations (made up in ultrapure water (MilliQ, Merck Millipore))—Triton X-100 (Merck, VIC, Australia), Ammonium hydroxide solution (Sigma Aldrich, NSW, Australia), and Sodium dodecyl sulfate (Sigma Aldrich, NSW, Australia). The treatments were carried out in 2L bottles (30% tissues) for five days with two solution changes per day. Tissue samples (< 50 mg wet weight) were collected for DNA analysis each day for each of the protocols (n = 3), followed by washes with ultrapure water for two days prior to storing the samples at -80 °C.

#### Homogenization

The decellularized liver ECM (d-LECM) was washed using milliQ water for 2 days at 4 °C with constant stirring. The washed tissues were homogenized using a blender (High setting, two min) and then frozen at -80 °C before being lyophilized for 3–4 days using a freeze-dryer (Christ, Osterode am Harz, Germany).

#### Solubilization

1 g of lyophilized d-LECM was solubilized using 100 mg of pepsin (Sigma Aldrich, NSW, Australia) in 100 ml of 0.01 M HCl and stirred at room temperature for 48 h. The pH of the digested tissue was approximately pH 4 – 5 and the reaction was stopped by increasing the pH to 8 using 0.1 M NaOH.

#### Methacrylation

Solubilized d-LECM (sd-LECM) was functionalized using methacrylation to obtain a photocrosslinkable hydrogel using previously developed methacrylation protocols^73^. Briefly, 0.6 g of Methacrylic anyhydride (MAAh) (Sigma Aldrich, NSW, Australia) was combined with 1 g of dECM to add methacryloyl groups on to the amine and hydroxyl groups of the solubilized liver dECM. The methacrylation was carried out for 24 h on ice with constant stirring (pH 8–9).

#### Dialysis

The methacrylated liver tissue or LivMA obtained was dialyzed against milliQ water using a dialysis tubing (MW cut-off: 2 kDa, Sigma-Aldrich, NSW, Australia) at 4 °C with constant stirring for a period of 5 days with water changes twice per day. This was done to remove insoluble MAAh and low molecular by-products^74^.

### Physicochemical characterization of LivMA

#### Decellularization

The extent of decellularization was assessed by analyzing DNA (Deoxyribonucleic acid) quantities obtained using Quant-iT™ PicoGreen™ Kit (Thermo Fisher Scientific, VIC, Australia) for each sample taken from the protocols at different time points. Routine Hematoxylin & Eosin (H&E) staining (Hematoxylin (POCD Scientific, NSW, Australia), t = 4 min; Eosin (Amber Scientific, WA, Australia), t = 1 min; Leica Autostainer XL, VIC, Australia) and immunohistochemical (IHC) staining were performed on 5 µm sections from fixed, paraffin-embedded fresh liver tissues and decellularized liver tissues from protocol I and protocol II. The expression of Collagen I (Col-1) (1:100, ab34710, Abcam) was studied by heat-mediated antigen retrieval (sodium citrate (pH 6), followed by 1 h primary antibody incubation. Rabbit IgG Isotype Control (Thermo Fisher Scientific, VIC, Australia) was used as the negative control. The staining was developed with the Envision + DualLink secondary HRP system (Agilent, VIC, Australia), 3,3′-dia-minobenzidine (DAB) chromogen substrate (Agilent, VIC, Australia) and counterstaining with Hematoxylin (t = 30 s, Sigma Aldrich, NSW, Australia).

#### Degree of functionalization

To determine the degree of functionalization (DOF), 2,4,6-Trinitrobenzenesulfonic acid (TNBS) (Sigma Aldrich, NSW, Australia) assay was used as described previously^57^. Briefly, solubilized liver ECM (non-functionalized) stock solution and LivMA (functionalized) stock solutions in 0.1 M NaHCO_3_ buffers were serially diluted to prepare 500 µg/mL, 250 µg/mL, 125 µg/mL, 62.5 µg/mL, and 31.25 µg/mL solutions. 0.01% (w/v) TNBS solution was added to each of the concentrations and incubated at 37 °C for 40 min–2 h and absorbance was read at 335 nm (n = 3–6).

### LivMA hydrogel fabrication

A stock solution of 1.75% (w/v) LivMA was obtained by dissolving lyophilized LivMA in sterile PBS at 4 °C for 24 h under constant rotation. Desired concentration of LivMA precursor solutions (0.5% (w/v), 0.75% (w/v)) were made by diluting the 1.75% (w/v) stock in PBS along with 0.15% (w/v) of photoinitiator lithium phenyl-2,4,6-trimethylbenzoylphosphinate (LAP) (Sigma Aldrich, NSW, Australia) (0.2 µm sterile filtered). The precursor solutions were added to wells of PTFE casting mold (1.5 mm height, 5 mm diameter) ((Queensland University of Technology Design and Manufacturing Centre (QUT DMC)) and photocrosslinked by exposure to 405 nm light in a visible light crosslinker (Gelomics, QLD, Australia).

### Mechanical testing of different concentrations of LivMA

LivMA hydrogels at 0.5% (w/v) and 0.75% (w/v) from protocols I and II were crosslinked a different crosslinking times—1 min, 2 min and 4 min and incubated in PBS at 37 °C overnight. Prior to the compression testing, the hydrogel surface area was imaged using stereomicroscope (Nikon, Melville, NY, USA) and measured using ImageJ software. In an unconfined compression test, the different gel groups submerged in 37 °C PBS-filled water bath were compressed using a 5848 Instron Microtester with a 5 N cell load (Instron, VIC, Australia) using a non-porous stainless-steel indenter at a strain rate of 0.01 mm/s. The compressive modulus was determined as the slope of the stress–strain curve from 10–15% strain (n = 6).

### Effective swelling

The effective swelling of the LivMA gels was calculated as the ratio of increase in hydrogel weight following overnight incubation in PBS at 37 °C on a shaker, to the initial hydrogel weight measured immediately following hydrogel preparation (n = 6).

### Cell culture

Immortalized Human Hepatocytes (IHH) (kindly provided by Professor Didier Trono from the Ecole Polytechnique Federale de Lausanne; EPFL) were cultured in DMEM/F-12 + GlutaMAX media supplemented with 10% Fetal Bovine Serum, 1 μM Dexamethasone and 1ug/ml Insulin at 37 °C, 5% CO_2_ (media and serum from Thermo Fisher Scientific, VIC, Australia; Dexamethasone and Insulin from Sigma Aldrich, NSW, Australia). Cells were resuspended in LivMA precursor solutions (0.5% (w/v) and 0.75% (w/v)) at a ratio of 0.5 × 10^6^ cells/mL and photocrosslinked by exposure to 405 nm for 1 min.

### Live/dead assay

The cell viability of IHH cells encapsulated in LivMA gels from days 1, 3 and 7 was analyzed by staining the gels with fluorescein diacetate/propidium Iodide (FDA/PI) (both reagents from Thermo Fisher Scientific, VIC, Australia) staining as described previously^73^. Briefly, cell-laden hydrogels were rinsed in PBS followed by 10 min incubation with 10 µg/mL of FDA and a 2 min incubation with 5 µg/mL stains live cells green and PI stains the nuclei red. The staining was visualized using a fluorescence stereomicroscope (Nikon, Melville, NY, USA).

### Metabolic assay

The metabolic activity of IHH cells encapsulated in the LivMA hydrogels was measured by incubating the gels with PrestoBlue™ Cell Viability Reagent for 5 h (Thermo Fisher Scientific, VIC, Australia) (9:1 of media:reagent). Relative fluorescence of the solution was measured (excitation at 544 nm, emission at 590 nm) on days 1, 3 and 7.

### Statistical analysis

Statistical analysis was performed using GraphPad Prism version 8.2.1. Unpaired t-test was performed to investigate the differences between protocols I and II after the 5 days of treatment. Two-way ANOVA followed by Tukey's multiple comparisons test was also used to study the differences in compressive moduli between the protocols at different GelMA concentrations and photocrosslinking times. Statistical significance is reported in the graphs using symbols—*p < 0.05, **p < 0.01, ***p < 0.001 and ***p < 0.0001.

## Supplementary Information

Supplementary Information.
