# Supplementary material for: Photocrosslinkable liver extracellular matrix hydrogels for the generation of 3D liver microenvironment models
Source: Sci Rep. 2021 Jul 30;11:15566. doi: 10.1038/s41598-021-94990-z (PMC8324893; doi:10.1038/s41598-021-94990-z)
Supplement: Supplementary file 1 — Supplementary Information. [file 41598_2021_94990_MOESM1_ESM.pdf]

Title

Photocrosslinkable liver extracellular matrix hydrogels for the generation of 3D liver microenvironment models

Authors

Akhilandeshwari Ravichandran[1,2,3,#, *], Berline Murekatete[1,#], Denise Moedder[1], Christoph Meinert[1,4,ⱡ], Laura J Bray[1,2,3,ⱡ]

Affiliations

1 Institute of Health and Biomedical Innovation, Queensland University of Technology (QUT), Kelvin Grove, Australia
2 ARC Training Centre for Cell and Tissue Engineering Technologies, Queensland University of Technology (QUT), Kelvin Grove, Australia
3 School of Mechanical, Medical and Process Engineering, Science and Engineering Faculty, Queensland University of Technology (QUT), Brisbane, Australia
4 Herston Biofabrication Institute, Metro North Hospital and Health Service, Herston, Australia

[#] These authors contributed equally.

[ⱡ] These authors share senior authorship.

Corresponding author:

Dr Akhilandeshwari Ravichandran

Supplementary Figures

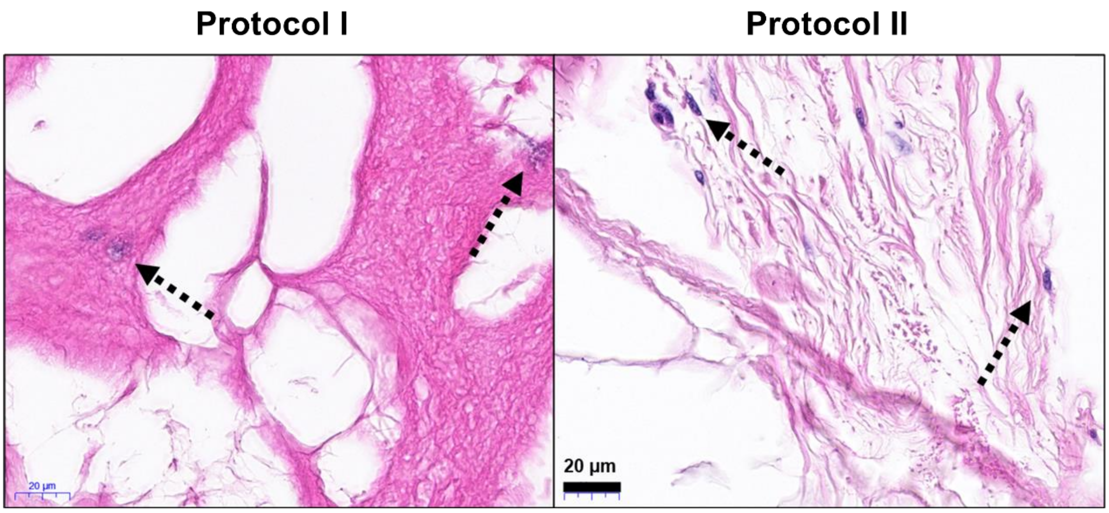

Supp Fig 1. H&E staining of decellularized tissues from Protocol I and Protocol II showing remnant nuclear content stained by hematoxylin (purple) pointed by dotted arrows.

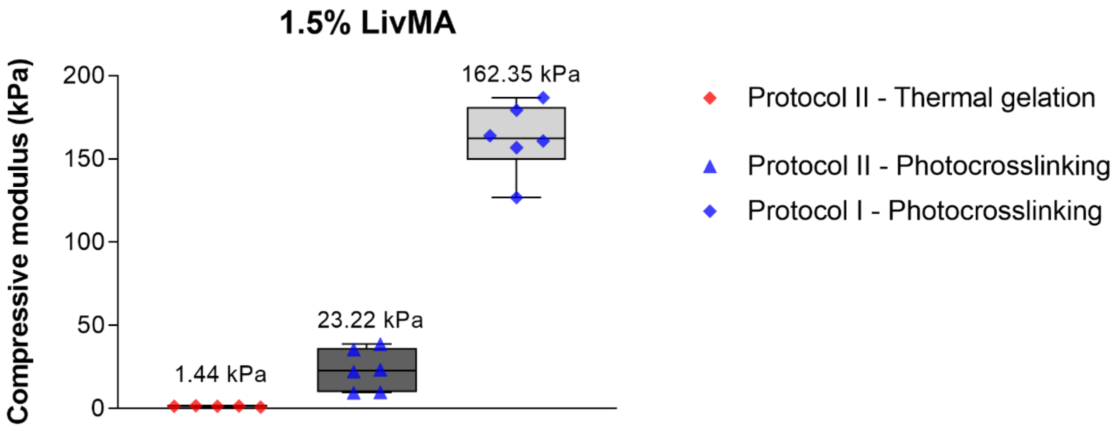

Supp Fig 2. Mechanical characterization of 1.5% LivMA hydrogels fabricated by thermal gelation (protocol II) and photocrosslinking (protocol I and II).

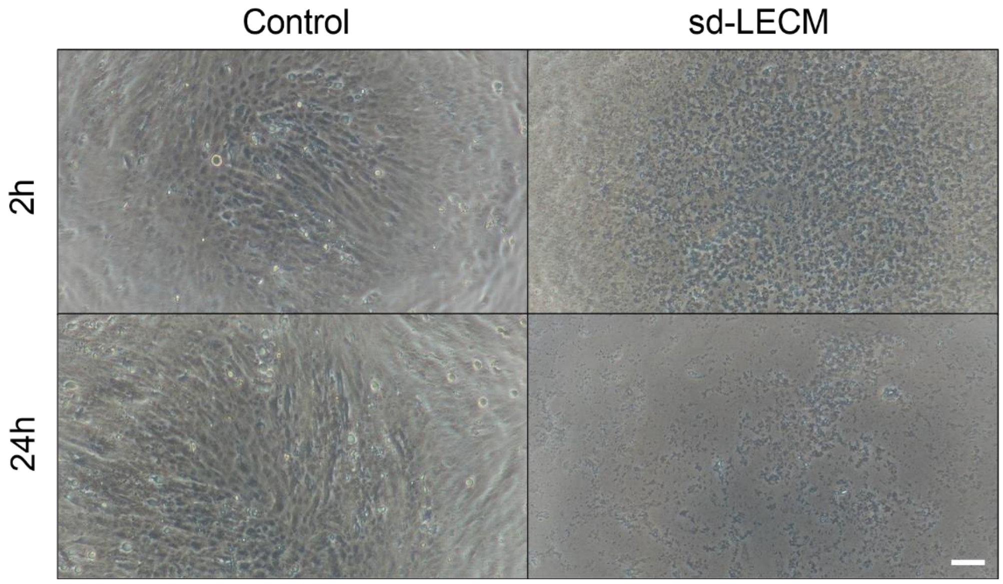

Supp Fig 3. 2D cytotoxicity assessment after 2h/24h of addition of sd-LECM solutions to IHH monolayer cultures (scale bar: 100 µm).
